# Massive Haemothorax Following CT-Guided Lung Biopsy Revealing Previously Undiagnosed Acquired Haemophilia A

**DOI:** 10.7759/cureus.111136

**Published:** 2026-06-19

**Authors:** Wajeeha Iqbal, Payal S Joshi, Santosh S Vijay, Mark S Maddock, Rahul S Chivate

**Affiliations:** 1 General Medicine, Worcestershire Health and Care NHS Trust, Redditch, GBR; 2 General Medicine, Worcestershire Acute Hospitals NHS Trust, Redditch, GBR; 3 Radiology, Worcestershire Acute Hospitals NHS Trust, Worcester, GBR; 4 Interventional Radiology, Worcestershire Acute Hospitals NHS Trust, Worcester, GBR

**Keywords:** acquired coagulopathy, haemophilia a, haemothorax, lung biopsy, pulmonary artery embolisation

## Abstract

CT-guided lung biopsy is a commonly performed procedure with recognised complications, including pneumothorax and pulmonary haemorrhage. Massive haemothorax following biopsy is rare and may indicate an underlying coagulopathy. We present the case of a 75-year-old man who developed a life-threatening haemothorax following a CT-guided biopsy of a left lower lobe lung lesion. Despite initial haemodynamic stability, the patient's clinical course required transfusion, pulmonary artery embolisation, chest drainage, and video-assisted thoracoscopic surgery (VATS). The development of spontaneous iliopsoas and quadriceps haematomas during admission prompted further haematological investigation, which revealed previously undiagnosed acquired haemophilia A (AHA). This report highlights the importance of recognising disproportionate bleeding following interventional procedures and maintaining a low threshold for investigating occult bleeding disorders. Early multidisciplinary involvement was essential in achieving a favourable outcome.

## Introduction

CT-guided lung biopsy is a frequently performed procedure that is associated with well-recognised complications, including pneumothorax and pulmonary haemorrhage. Pneumothorax is the most frequent complication, with a pooled incidence of approximately 25.9%, while pulmonary haemorrhage occurs in 4-27% of cases, depending on definition and technique. Haemothorax is a far less common sequela; large series and meta-analyses report an overall incidence of 1.8-3.1%, with severe or life-threatening haemorrhagic complications - encompassing massive haemothorax, severe haemoptysis, and significant pulmonary haemorrhage - occurring in fewer than 0.1-0.3% of procedures. Massive haemothorax following biopsy is rare and may indicate an underlying coagulopathy.

We present the case of a 75-year-old man who developed a life-threatening haemothorax following CT-guided biopsy of a left lower lobe lung lesion. Despite initial haemodynamic stability, the patient's clinical course required transfusion, pulmonary artery embolisation, chest drainage, and video-assisted thoracoscopic surgery (VATS). The development of spontaneous iliopsoas and quadriceps haematomas during admission prompted further haematological investigation, which revealed previously undiagnosed acquired haemophilia A (AHA).

AHA is a rare autoimmune bleeding disorder caused by autoantibodies against coagulation factor VIII. Its annual incidence is estimated at around 1.0-1.5 cases per million in the general population, increasing significantly with age to approximately three to four cases per million per year among individuals aged 60 years and above. The condition predominantly affects older adults, with a median age at diagnosis of about 73-75 years in large European registry studies, and shows a slight male predominance outside pregnancy-associated cases. In roughly half of patients, no underlying cause is identified, and the condition is therefore classified as idiopathic.

Among cases with an identifiable cause, malignancy and autoimmune disease are the most common associated conditions, each accounting for approximately 11-14% of patients in registry studies. Less frequently, AHA has been linked to pregnancy, drug exposure, and various dermatological disorders. The malignancies most often reported include both solid organ tumours and haematological cancers, while the autoimmune conditions most commonly implicated are rheumatoid arthritis and systemic lupus erythematosus. Patients usually present with spontaneous bleeding involving soft tissues, muscles, or mucosal surfaces, and diagnosis can be difficult in the absence of a prior bleeding history. This case report underscores the importance of recognising excessive or disproportionate bleeding following interventional procedures and maintaining a low threshold for investigating underlying bleeding disorders. Early involvement of a multidisciplinary team was crucial in achieving a favourable clinical outcome.

## Case presentation

A 75-year-old man with a background of coeliac disease and prostate adenocarcinoma (pathological stage pT2aN0, Gleason score 3+4=7) was under routine surveillance following a radical retropubic prostatectomy performed in February 2015. Surgical margins were negative, and he was maintained on regular prostate-specific antigen (PSA) monitoring. He presented with a four-month history of recurrent chest infections. Chest radiography demonstrated persistent parenchymal changes, prompting further investigation with CT imaging of the thorax and abdomen. Imaging revealed a left-sided renal mass suspicious for a primary renal malignancy and a left lower lobe nodular lesion measuring 2.1 × 2.7 × 3.4 cm, suspicious for either metastatic disease or a primary lung malignancy. Additional small indeterminate pulmonary nodules were identified within the left lung and right upper lobe.

Following multidisciplinary team (MDT) discussion, a CT-guided biopsy of the left lower lobe lesion was arranged (Figure 1) [1,2,3]. Pre-procedural assessment did not include routine coagulation screening. The patient had no personal or family history of bleeding disorders, was not taking anticoagulant or antiplatelet therapy, and there were no clinical indicators suggestive of an underlying haemostatic abnormality at the time of the procedure. Coagulation studies, including activated partial thromboplastin time (APTT), were therefore performed only after the bleeding complication had become apparent.

**Figure 1:**
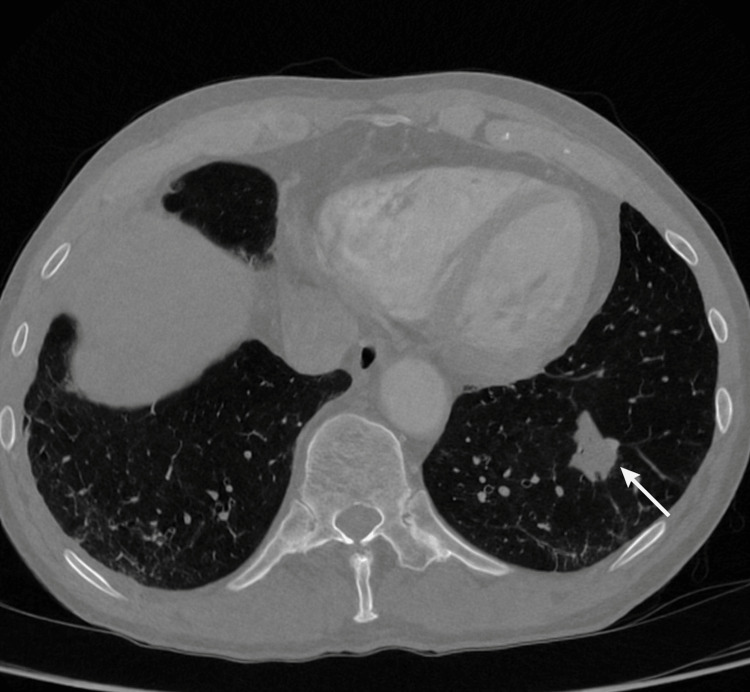
Axial non-contrast CT scan of the lung demonstrating a lesion in the left lower lobe (arrow) CT: computed tomography

Shortly after the procedure, the patient collapsed while undergoing a post-biopsy chest radiograph, prompting an emergency medical team review. Initial chest radiography performed approximately one hour after the procedure did not demonstrate any significant abnormality (Figure 2). However, laboratory investigations revealed a marked drop in haemoglobin from 115 g/L to 61 g/L. Coagulation studies demonstrated a prothrombin time (PT) of 13 seconds, an international normalised ratio (INR) of 1.1, and an APTT of 53 seconds. Renal function was normal. Full blood count results are summarised in Table 1.

**Table 1:** Full blood count results

Parameter	Result	Unit	Reference range	Interpretation
Haemoglobin	61	g/L	135–180	Low
White blood cells (WBC)	7.8	×10⁹/L	4.0–11.0	Normal
Platelets (PLT)	335	×10⁹/L	150–400	Normal
Red blood cells (RBC)	1.93	×10¹²/L	4.50–6.50	Low
Haematocrit (HCT)	0.181	L/L	0.40–0.54	Low
Mean corpuscular volume (MCV)	94.0	fL	78.0–96.0	Normal
Mean corpuscular haemoglobin (MCH)	31.9	pg	28.0–32.0	Normal
Red cell distribution width (RDW)	15.9	%	11.5–16.0	Normal
Neutrophils	4.4	×10⁹/L	2.0–7.5	Normal
Lymphocytes	2.2	×10⁹/L	1.0–4.0	Normal
Monocytes	0.7	×10⁹/L	0.2–1.0	Normal
Eosinophils	0.4	×10⁹/L	0.0–0.4	Upper limit of normal
Basophils	0	×10⁹/L	0.0–0.1	Normal

**Figure 2:**
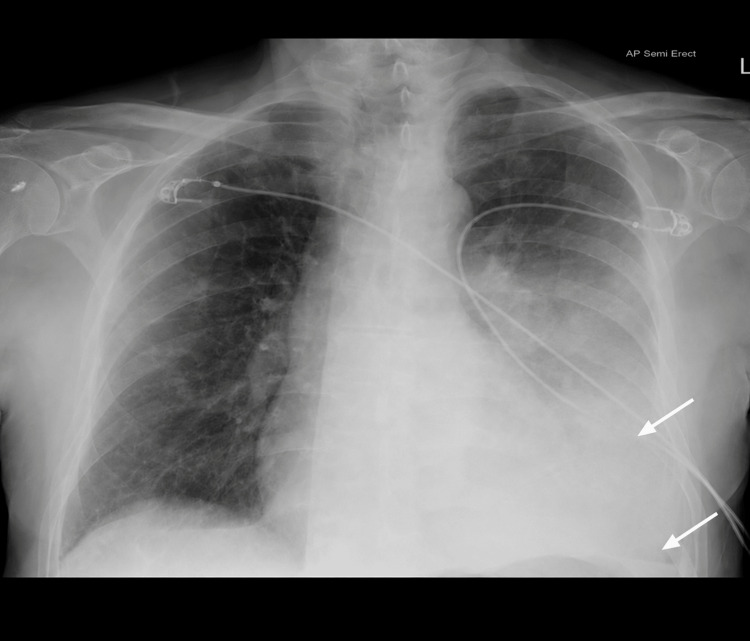
Post-biopsy chest radiograph demonstrating haziness within the left hemithorax, more prominent in the lower zone, with mild blunting of the costophrenic angle (arrowheads)

The patient received two units of packed red blood cells. Given the significant haemoglobin drop and worsening left-sided pleuritic chest pain, urgent contrast-enhanced CT of the thorax was performed. This demonstrated a large left haemothorax with active contrast extravasation arising from a branch of the left lower lobe pulmonary artery along the biopsy tract (Figure 3).

**Figure 3:**
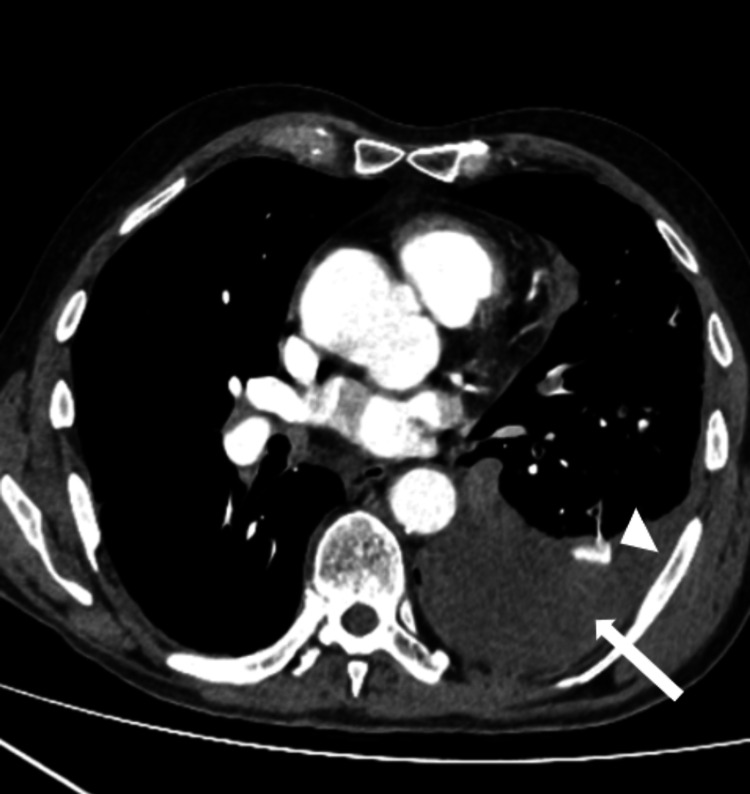
Post-biopsy contrast CT showing significant left haemothorax (arrow) with active extravasation of contrast from lower lobe pulmonary arterial branch (arrowhead) CT: computed tomography

At this stage, the patient remained haemodynamically stable with satisfactory oxygen saturation and was initially managed conservatively with tranexamic acid, vitamin K, blood product support, and close monitoring in the ICU. Due to concerns regarding ongoing bleeding, he was transferred to a tertiary centre for consideration of embolisation.

Later the same evening, the patient became hypotensive and developed worsening hypoxia, requiring up to 15 litres of supplemental oxygen. The major haemorrhage protocol was activated. At this point, we chose to hold off on inserting a chest drain. The CT scan had already shown active bleeding from a pulmonary artery branch into the pleural space, and we were concerned that draining the chest too early could make things significantly worse. The blood that had collected around the lung was, in effect, acting as a natural tamponade - pressing against the bleeding vessel and helping to slow the haemorrhage. Removing that blood before the bleeding source had been dealt with risked destabilising the patient rapidly and unpredictably.

We therefore prioritised stopping the bleeding at its source first. Once the interventional radiology team had successfully embolised the responsible lower lobe pulmonary artery branches, and we were satisfied that haemostasis (Figures 4, 5) had been achieved, a left-sided chest drain was inserted. This drained approximately 3 litres of blood and was well tolerated haemodynamically. In our experience, and consistent with established principles in managing traumatic haemothorax with active arterial injury, securing the bleeding source before decompressing the pleural space is a critical sequencing decision - one that we feel is worth highlighting for clinicians who may encounter a similar scenario following CT-guided lung biopsy.

**Figure 4:**
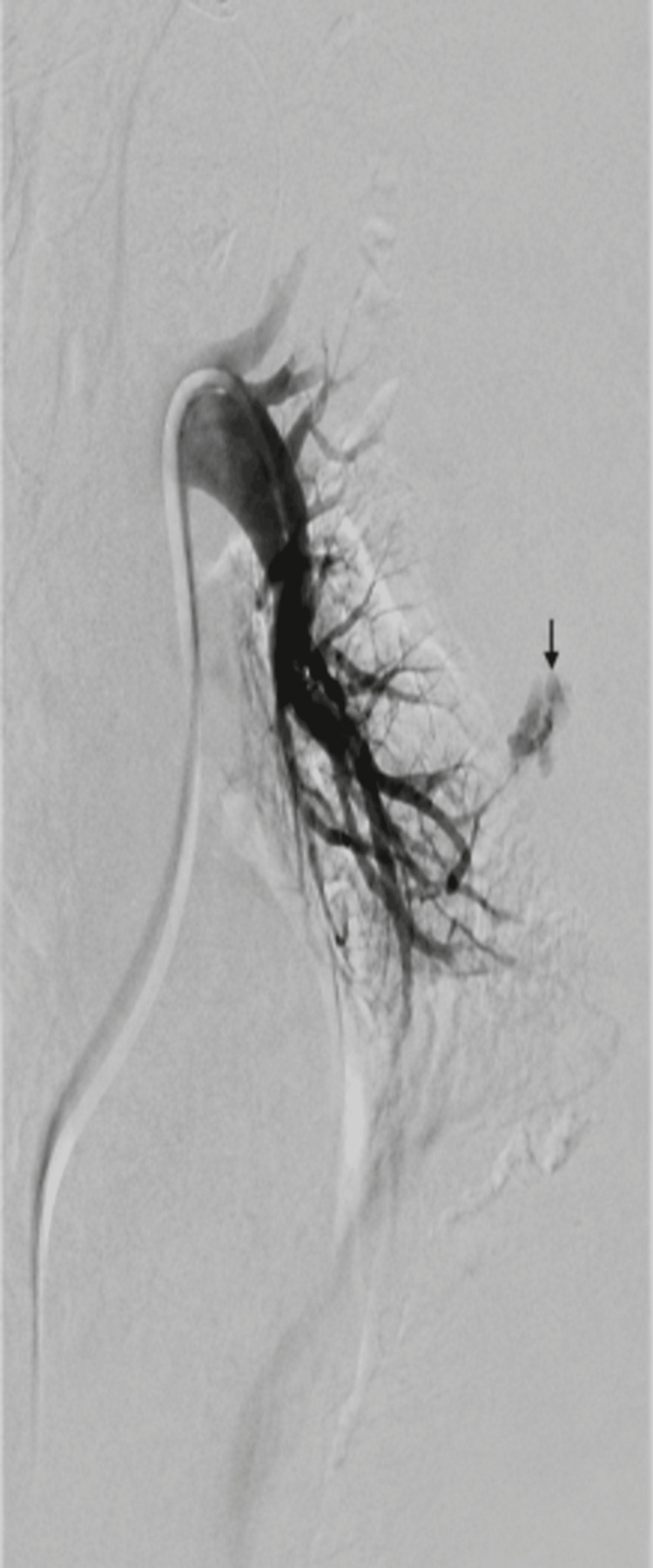
Selective pulmonary angiography demonstrating active contrast extravasation from a lower lobe pulmonary arterial branch (arrow), indicating ongoing haemorrhage

**Figure 5:**
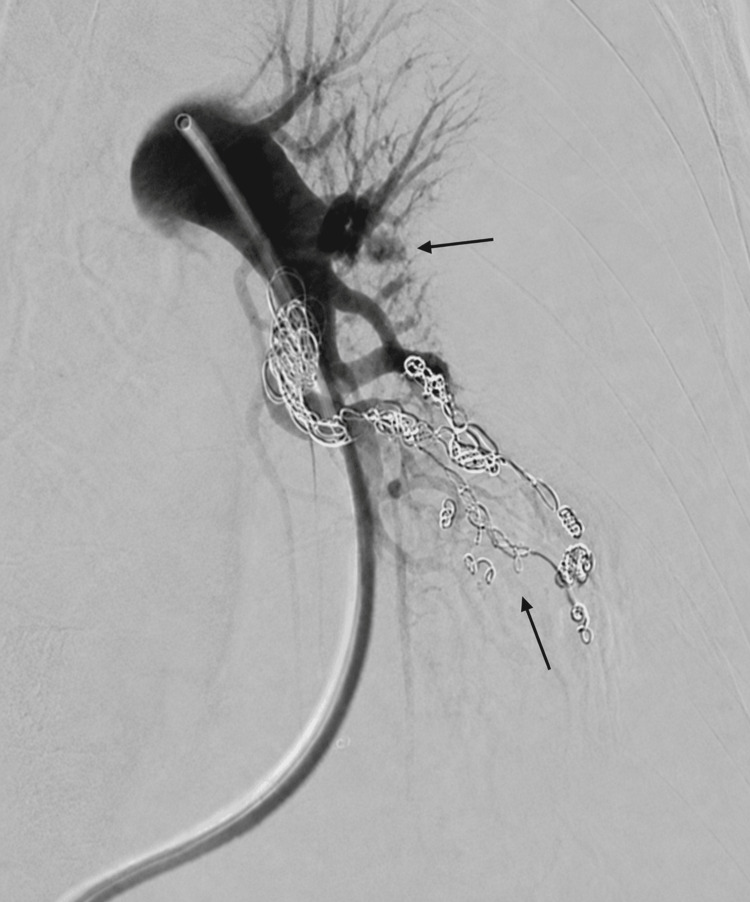
Pulmonary angiography demonstrating successful coil embolisation for haemostasis (arrows)

Post-embolisation CT imaging demonstrated successful occlusion of the bleeding lower lobe pulmonary arterial branches with no further active contrast extravasation. Residual left-sided haemothorax was present; however, the interval reduction in active haemorrhage confirmed the technical success of the procedure. The patient subsequently underwent left VATS washout for definitive management of the retained haemothorax.

During admission, the patient developed acute right lower limb weakness. Imaging demonstrated large haematomas involving the right iliopsoas and quadriceps muscles (Figure 6), and electromyography confirmed right femoral nerve dysfunction secondary to compressive neuropathy. Further haematological investigations confirmed acquired haemophilia A with markedly reduced factor VIII activity (Table 2). The patient was treated with recombinant porcine factor VIII, factor eight inhibitor bypassing activity (FEIBA), and high-dose corticosteroids under specialist haematology guidance [4]. Factor VIII levels subsequently normalised, and inhibitor levels became undetectable following treatment. Once clinically stable, he was stepped down from intensive care to the haematology ward and was later discharged with ongoing outpatient follow-up.

**Table 2:** Coagulation and haemostasis profile PT: prothrombin time; INR: international normalised ratio; APTT: activated partial thromboplastin time

Parameter	Result	Unit	Reference range	Interpretation
Factor XI assay	81.7	IU/dL	65–150	Normal
Factor VIII assay	0.9	IU/dL	50–150	Markedly low
Factor IX assay	129.5	IU/dL	65–150	Normal
Fibrinogen assay	6.89	g/L	2.0–6.0	Elevated
Von Willebrand factor antigen	344.3	IU/dL	66–176	Elevated
Ristocetin co-factor	259.5	IU/dL	61–240	Elevated
PT	13	Sec	9–14	Upper limit of normal
PT ratio/INR	1.1		0.8–1.2	Upper limit of normal
APTT	53	Sec	24–37	Prolonged
APTT ratio	2.8		0.8–1.2	Prolonged

**Figure 6:**
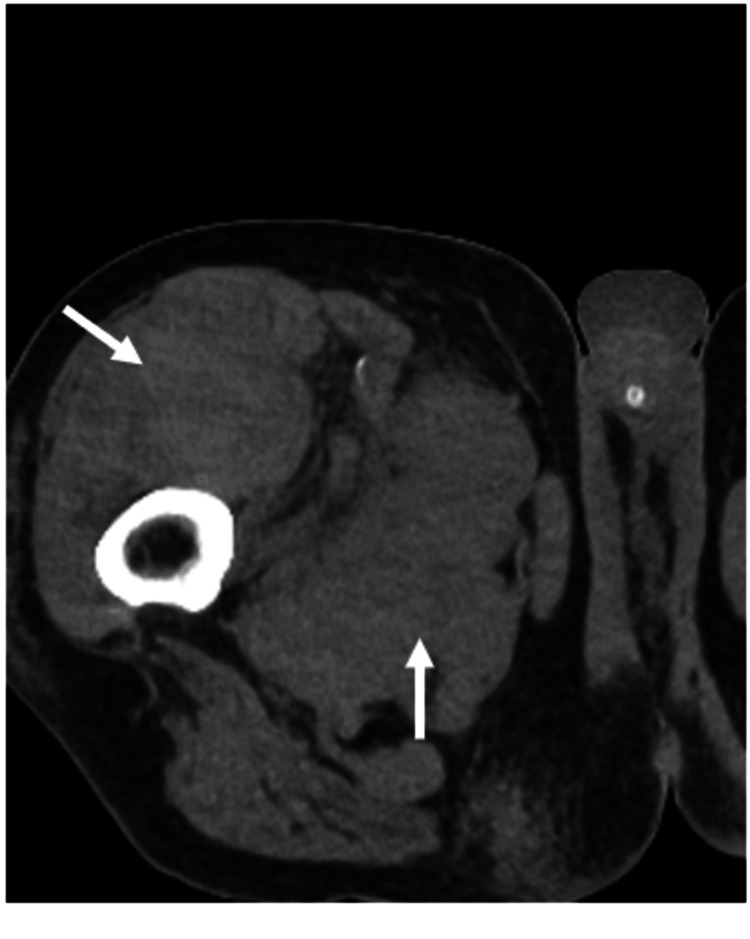
CT scan showing haematoma in thigh/groin (arrows) CT: computed tomography

Histopathological analysis of the lung biopsy demonstrated benign lung tissue with fibrosis and dense chronic lymphoplasmacytic inflammatory infiltrate. No evidence of malignancy or granulomatous disease was identified.

## Discussion

CT-guided lung biopsy is an established diagnostic procedure with a relatively low rate of major complications [1,2]. Pneumothorax and mild pulmonary haemorrhage are the most frequently encountered adverse events, while severe haemothorax remains rare [3]. Although uncommon, injury to pulmonary arterial branches may result in life-threatening intrathoracic bleeding requiring urgent intervention [5,6]. In our patient, the extent of bleeding was disproportionate to the procedure and prompted further investigation for an underlying bleeding disorder. AHA is a rare but serious autoimmune condition caused by autoantibodies directed against factor VIII [4]. It predominantly affects older adults and may occur in association with autoimmune disease or malignancy, or may arise idiopathically. Patients typically present with spontaneous soft tissue, intramuscular, or mucosal bleeding rather than haemarthrosis, which is more characteristic of congenital haemophilia. This distinction is clinically important, as the diagnosis may not be immediately suspected in patients without a prior bleeding history.

Several features of this patient's profile are well-recognised risk factors for the development of AHA and should heighten clinical suspicion when disproportionate bleeding is encountered. The patient's age of 75 years is highly relevant; AHA is predominantly a disease of older adults, and the incidence in those aged 60 and over is estimated at three to four cases per million per year. His background of prostate adenocarcinoma is also a recognised association, with malignancy accounting for approximately 11-15% of cases with an identifiable underlying aetiology. Furthermore, coeliac disease, as an immune-mediated condition, represents a form of chronic immune dysregulation that may predispose to the development of pathological autoantibodies, including those directed against factor VIII. In retrospect, the convergence of advanced age, underlying malignancy, and an autoimmune background placed this patient within a high-risk demographic for AHA, even in the absence of a prior bleeding history.

The diagnosis was not suspected before the procedure, which is consistent with the typical clinical trajectory of the condition. AHA characteristically presents in individuals with no prior personal or family history of bleeding, and coagulation screening is not routinely performed before CT-guided lung biopsy in the absence of clinical risk factors [7]. The key laboratory clue - a prolonged APTT with a normal PT and normal platelet count, first identified after the haemorrhagic complication - is the hallmark haematological signature of the condition, reflecting isolated impairment of the intrinsic coagulation pathway. The subsequent development of spontaneous iliopsoas and quadriceps haematomas further raised suspicion for an acquired coagulation disorder and led to definitive factor assay testing. Clinicians encountering bleeding that appears disproportionate to the procedure performed, particularly in older patients with malignancy or immune-mediated disease, should maintain a low threshold for urgent haematological review [8,9,10].

Management of major bleeding in AHA is particularly challenging and frequently requires a coordinated multidisciplinary approach involving haematology, interventional radiology, thoracic surgery, and critical care teams [4]. In this case, successful haemostasis was achieved through pulmonary artery embolisation, blood product support, haemostatic therapy, and subsequent surgical washout [5]. The sequence of interventions was critical in stabilising the patient and preventing further deterioration. This case also highlights the complexity of decision-making regarding chest drain insertion in the setting of an actively bleeding haemothorax. Premature decompression risks reducing the tamponade effect and may exacerbate ongoing haemorrhage. Delaying drainage until haemostasis had been achieved through embolisation allowed safer subsequent evacuation of the retained haemothorax, and this staged approach - source control before pleural decompression - is consistent with established principles in the management of traumatic and iatrogenic haemothorax with active arterial injury.

Furthermore, this case underlines the importance of careful pre-procedural assessment and maintaining a high index of suspicion for occult bleeding disorders when post-procedural complications appear disproportionate to the intervention performed. Significant or unexplained bleeding following minimally invasive procedures should always prompt consideration of an underlying coagulopathy, particularly in older patients with no prior bleeding history [8,9,10].

## Conclusions

This case report described a rare but life-threatening haemothorax following CT-guided lung biopsy, which ultimately led to the diagnosis of previously unrecognised AHA. Significant or disproportionate bleeding following minimally invasive procedures should prompt consideration of an underlying occult coagulopathy, particularly in older patients with no prior bleeding history and unexplained prolongation of APTT. Early recognition, prompt escalation, and coordinated multidisciplinary management are essential for achieving a favourable outcome in such cases.
